# Protection against Oxidative Stress in Beta Thalassemia/Hemoglobin E Erythrocytes by Inhibitors of Glutathione Efflux Transporters

**DOI:** 10.1371/journal.pone.0055685

**Published:** 2013-01-31

**Authors:** Chatchai Muanprasat, Chokdee Wongborisuth, Nutthapoom Pathomthongtaweechai, Saravut Satitsri, Suradej Hongeng

**Affiliations:** 1 Research Center of Transport Protein for Medical Innovation, Department of Physiology, Faculty of Science, Mahidol University, Bangkok, Thailand; 2 Research Center, Faculty of Medicine, Ramathibodi Hospital, Mahidol University, Bangkok, Thailand; 3 Department of Pediatrics, Faculty of Medicine, Ramathibodi Hospital, Mahidol University, Bangkok, Thailand; University of Texas MD Anderson Cancer Center, United States of America

## Abstract

In beta thalassemia/hemoglobin E (Hb E), abnormally high levels of oxidative stress account for accelerated senescence and increased destruction of erythrocytes. The present study aimed to investigate the role of glutathione efflux transporters, namely cystic fibrosis transmembrane conductance regulator (CFTR) and multidrug resistance-associated protein 1 (MRP1), in the control of glutathione levels and protection against oxidative challenges in beta thalassemia/Hb E erythrocytes. We found that CFTR protein was expressed in the erythrocytes of beta thalassemia/Hb E patients. Treatments with GlyH-101 (50 µM), a small molecule CFTR inhibitor, and MK571 (50 µM), an MRP1 inhibitor, reduced H_2_O_2_-induced free radical generation in the erythrocytes by ∼80% and 50%, respectively. Furthermore, combined treatment with GlyH-101 and MK571 completely abolished the induction of reactive oxygen radicals. Increased oxidative stress in the erythrocytes following H_2_O_2_ challenges was accompanied by a decrease in intracellular level of reduced glutathione (GSH), which was prevented by treatments with GlyH-101 and MK571. CMFDA-based assays revealed that GlyH-101 and MK571 reduced H_2_O_2_-induced glutathione efflux from the erythrocytes by 87% and 66%, respectively. Interestingly, H_2_O_2_-induced osmotic tolerance of erythrocytes, a sign of erythrocyte aging, was ameliorated by treatment with GlyH-101. Our study indicates that oxidative stress induces glutathione efflux via CFTR and MRP1 in beta thalassemia/Hb E erythrocytes. Pharmacological inhibition of glutathione efflux represents a potential therapy to delay aging and premature destruction of erythrocytes in beta thalassemia/Hb E.

## Introduction

Thalassemia is a hematological genetic disorder caused by deficiency of alpha or beta chains of hemoglobins, which are known as alpha or beta thalassemia, respectively [1], [2]. Beta thalassemia/hemoglobin E (Hb E) is a form of beta thalassemia commonly found in South East Asia including Thailand [3], [4]. In this disease, the synthesis of beta globin chain is insufficient, causing aggregations of excessive unpaired alpha globin chains [5], [6]. The alpha chain aggregates could produce reactive oxygen species, leading to oxidative stress-induced red blood cell senescence characterized by externalization and release of phosphatidylserine [6]. The oxidation-damaged erythrocytes are subject to premature phagocytic destruction in the spleen and, therefore, have a short life span in circulation [7]. These pathological events underline severe anemia and splenomegaly observed in beta thalassemia/Hb E patients [7].

Reduced glutathione (GSH) is an important endogenous antioxidant in all cell types including erythrocytes [8]. Levels of GSH inside the cells are tightly regulated by the rate of GSH synthesis and GSH efflux via membrane transporters, namely multidrug resistance-associated protein (MRP), cystic fibrosis transmembrane conductance regulator (CFTR), and organic anion transporting polypeptide [8]. Among MRPs, MRP 1, MRP 2, MRP4 and MRP 5 can transport GSH and other glutathione conjugates including oxidized glutathione (GSSG) [8]. In addition to serving as chloride channels, CFTR plays an important role in exporting GSH and glutathione conjugates from airway epithelial cells into airway surface liquid, which provides protection of the airways from oxidative damage during infection and inflammation [9]–[11]. Indeed, effluxes of GSH and GSSG precede oxidative stress-induced apoptosis of several cell types, including astrocytes, endothelial cells, epithelial cells and erythrocytes [12]–[16]. Pharmacological blockage and genetic ablation of glutathione efflux transporters have been shown to prevent oxidative stress-induced apoptosis in renal epithelial cells by preventing effluxes of GSH and GSSG, which, in turn, reduce production of reactive oxygen species (ROS) [17].

**Figure 1:**
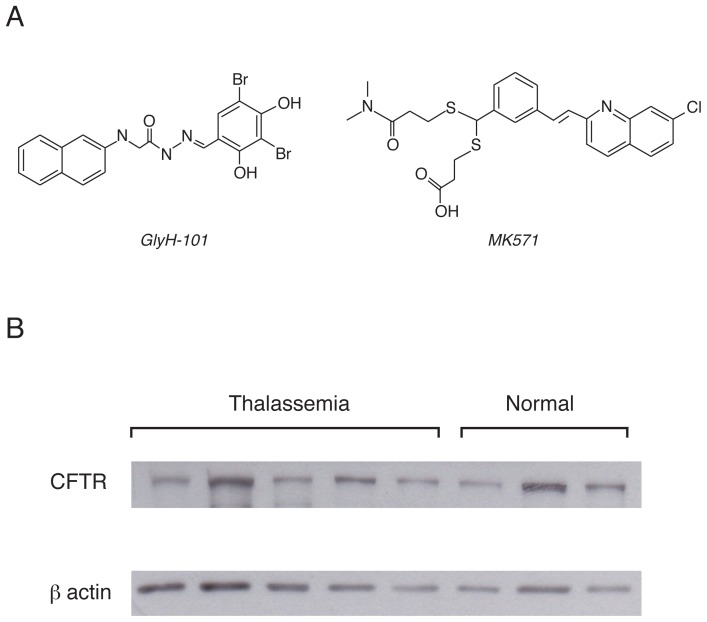
Chemical structures of inhibitors of glutathione efflux transporters and CFTR expression in human erythrocytes. (A) Chemical structures of GlyH-101, a CFTR inhibitor, and MK571, a MRP1 inhibitors. (B) Expression of CFTR in erythrocytes of beta thalassemia/Hb E patients and normal healthy subjects.

GlyH-101 and MK571 (Fig. 1A) are well-characterized inhibitors of CFTR and MRP, respectively. GlyH-101 is a CFTR inhibitor discovered by high-throughput screening [18]. Previous studies have shown that GlyH-101 blocks CFTR by occluding the external pore of CFTR and that GlyH-101 administration prevents cholera toxin-induced intestinal fluid secretion in mouse closed loop models [18]. MK571 is an orally active MRP inhibitor that has been used in the management of asthma [19]. Since MRPs (especially MRP1) and CFTR are expressed in erythrocytes and mediate oxidative stress-induced glutathione efflux in several types of cells [8], [15], [20], [21], we, therefore, hypothesized that pharmacological inhibition of these two glutathione transporters may reduce oxidative stress and its consequences in the erythrocytes. Due to an abundance of patients and their high oxidative stress burden [22], erythrocytes obtained from beta thalassemia/Hb E patients were used in this study. Herein, we demonstrated that treatments with GlyH-101 and MK571 attenuated H_2_O_2_-induced ROS production and osmotic tolerance, a sign of erythrocyte aging, in erythrocytes of beta thalassemia/Hb E patients by preventing glutathione effluxes.

## Materials and Methods

### Blood samples and volunteers

Blood was collected from beta thalassemia/Hb E patients at the thalassemia clinic, Ramathibodi Hospital. A total of 39 beta thalassemia/Hb E patients were studied. Nineteen patients were male and 20 were female. Age of the subjects ranged from 11 to 21 years old with a median of 15 years. Serum ferritin levels of the subjects ranged from 1,120 to 6,045 ng/mL with a median of 4,200 ng/mL. Eighteen of 39 had splenectomy. All patients had hepatosplenomegaly unless they had splenectomy and were transfusion-dependent. The patients did not receive blood transfusion within 1–2 months and did not take any medications for at least 1 week prior to blood sample collections except iron chelating agents and folic acid supplement. All of these patients were defined as severe thalassemia patients based on criteria from Thalassemia International Federation (TIF); 1) onset of disease at the first 3 years of life 2) hepatosplenomegaly 3) frequent packed red cell transfusion 4) hemoglobin level prior transfusion ≤8 mg/dL [3]. The study protocol was approved by the Ramathibodi Hospital Ethics Committee. Written informed consents were obtained in accordance with the Declaration of Helsinki.

### Western blot analysis of CFTR

After centrifugation of the erythrocyte homogenates (10,000 g) for 20 min, protein concentrations were determined by Lowry method [23], and the samples were separated by SDS-PAGE and transferred onto a nitrocellulose membrane. The membrane was then incubated for 1 h with 5% non-fat milk and overnight with rabbit antibodies to CFTR and β actin (Cell Signaling Technology, Danvers, MA, USA.), followed by three washes with Tris-Buffered Saline Tween-20 (TBST) and incubation for 1 h with horseradish peroxidase-conjugated goat antibody to rabbit immunoglobulin G (Cell Signaling Technology, Danvers, MA, USA.) at room temperature. The immunoblot was detected by using chemiluminescence assay kits (PerkinElmer, Waltham, MA, USA).

### Measurements of oxidative stress

Blood was centrifuged at 1,500 g at 4°C for 10 min and pellets were washed three times with phosphate-buffered saline (PBS). Cells were then loaded with 2′, 7′-dichlorodihydrofluorescin diacetate (DCFH-DA; 0.4 mM)) for 30 min, washed three times with PBS and incubated at 37°C for 1 h with DMSO, GlyH-101 (50 µM), MK571 (50 µM), H_2_O_2_ (10 mM) plus DMSO, H_2_O_2_ plus GlyH-101, H_2_O_2_ plus MK571 or H_2_O_2_ plus GlyH-101 and MK571. Reactive oxygen species were measured by flow cytometry analysis of 15,000 erythrocytes using mean fluorescence intensity at excitation wavelength of 490 nm and emission wavelength of 530 nm (Coulter EPICS XL, Beckman Coulter Inc., Indianapolis, IN, USA.).

### Measurements of intracellular reduced glutathione (GSH)

Intracellular reduced glutathione (GSH) contents were measured using protocol modified from Rahman et al [24]. Briefly, blood was centrifuged at 1,500 g at 4°C for 10 min and pellets were washed three times with PBS. Cells were then incubated at 37°C for 1 h with DMSO, GlyH-101 (50 µM), MK571 (50 µM), H_2_O_2_ (10 mM) plus DMSO, H_2_O_2_ plus GlyH-101, H_2_O_2_ plus MK571 or H_2_O_2_ plus GlyH-101 and MK571. After centrifugation and removal of the supernatant, erythrocyte pellets were then resuspended with 5% metaphosphoric acid working solution, mixed and centrifuged at 3,000 g at 4°C for 10 min. The upper clear aqueous layer was collected for GSH assays according to the protocol suggested by Rahman et al [24].

### CMFDA-based assay of glutathione efflux

5-chloromethylfluorescein diacetate (CMFDA) efflux measurements were previously used to quantify glutathione efflux via both MRPs and CFTR [12], [25]. CMFDA is nonfluorescent and passively accumulated in the cells because of its lipophilic property. Inside the cells, acetate residues are cleaved off by intracellular esterases, releasing the fluorescent and cell-impermeable product CMF. CMF then reacts with glutathione to form a fluorescent conjugate MF-SG, which is a substrate of glutathione transporters such as MRPs and CFTR. In brief, after centrifugation of erythrocytes at 1,500 g at 4°C for 10 min, the supernatants were discarded and the pellets were washed three times with PBS by centrifugation. Erythrocytes (500,000 cells/ml) were then loaded with CMFDA (1 µM in PBS) at 37°C for 1 h with continuous shaking. After three cycles of cell washing with CMFDA-free PBS, erythrocytes were exposed to PBS containing vehicle (basal condition), H_2_O_2_ (10 mM), H_2_O_2_ plus GlyH-101 (50 µM) or H_2_O_2_ plus MK571 (50 µM). Glutathione efflux was estimated by subtracting the amount of MF-SG in extracellular medium prior to treatments from that at 1 h after treatments. MF-SG was detected by a spectrofluorometer (wavelength of 495/515, JASCO FP-6200, Essex, UK). Extracellular cell-free heme was measured to correct for glutathione loss due to hemolysis according to Barrand et al [26].

### Osmotic tolerance test

Blood samples were centrifuged at 1,500 g at 4°C for 10 min. The supernatants were discarded and the pellets were washed three times with PBS at 1,500 g at 4°C for 10 min. The cell pellets were divided into four portions. The first three portions were incubated with PBS containing H_2_O_2_ (10 mM) with or without GlyH-101 (50 µM) at 37°C for 3 h in a shaking incubator. After centrifugation and removal of supernatant, cells were exposed to hypotonic solution (85% PBS) for 5 min and centrifuged at 1,500 g at 4°C for 10 min. The last portion of erythrocyte pellets was dissolved in distilled water to induce complete hemolysis. Absorbance of the supernatants from all groups was measured at 540 nm. Data were expressed as percentage of complete hemolysis.

### Statistical analysis

Data were presented as means ± S.E. Statistical analysis was made by Student's t-test or one-way analysis of variance (ANOVA), where appropriate. Statistical significance was considered when p<0.05.

## Results

### Effects of GlyH-101 on ROS production and glutathione levels in erythrocytes

Previous studies have shown that CFTR is expressed in red blood cells of healthy human [21]. In order to confirm the expression of CFTR protein in the erythrocytes of patients with beta thalassemia/Hb E, western blot analysis of the erythrocyte homogenates was performed. As depicted in Fig. 1B, CFTR proteins were detected in all 5 thalassemia patients. As expected, expression of CFTR proteins was also found in the erythrocytes from normal subjects. No significant differences in CFTR expression between the two groups were observed.

**Figure 2:**
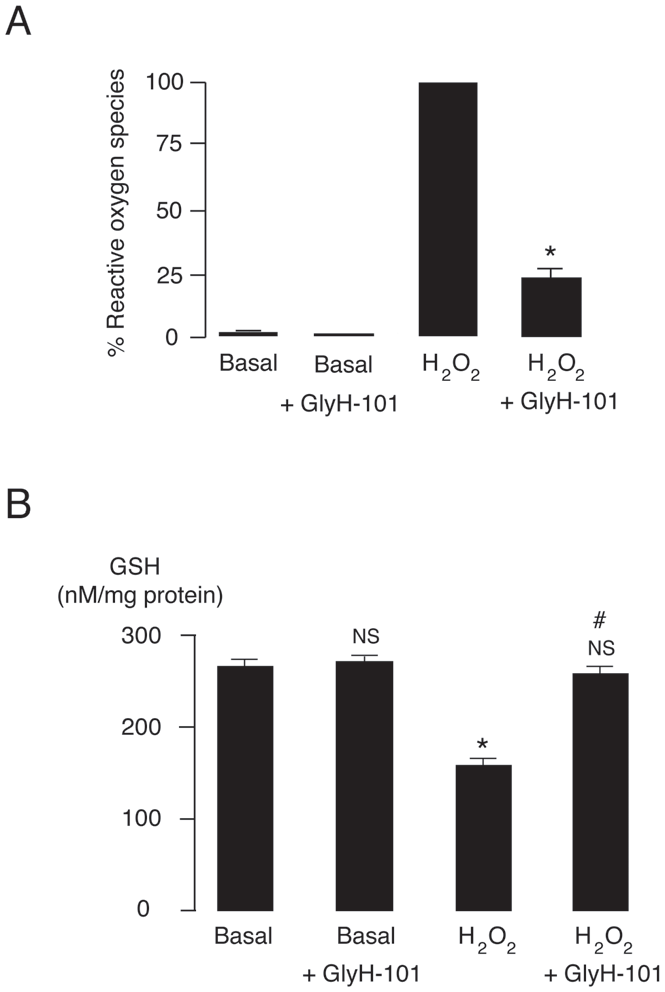
Anti-oxidative properties of GlyH-101 in erythrocytes of beta thalassemia/Hb E patients. (A) Amelioration by GlyH-101 of free radical production induced by H_2_O_2_ exposure. Erythrocytes loaded with DCFH-DA were incubated for 1 h without (basal) or with H_2_O_2_ (10 mM) in the presence or absence of GlyH-101 (50 µM). Reactive oxygen species were measured by flow cytometry analysis at excitation wavelength of 490 nm and emission wavelength of 530 nm. Data were expressed as means ± S.E. *, p<0.001 compared with H_2_O_2_-treated group (n = 12). (B) Effect of GlyH-101 on intracellular glutathione levels. Erythrocytes were treated for 1 h without (basal) or with H_2_O_2_ (10 mM) in the presence or absence of GlyH-101 (50 µM) before measurements of GSH levels. Data were expressed as means ± S.E. NS, non-statistical significant difference from basal control; *, p<0.01 compared with basal control; #, p<0.01 compared with H_2_O_2_-treated group (n = 12).

To determine whether CFTR plays a role in the regulation of oxidative stress, the effect of GlyH-101 (50 µM) on H_2_O_2_-induced ROS production was investigated using flow cytometry analysis of DCFH-DA staining. As shown in Fig. 2A, GlyH-101 had virtually no effect on basal ROS levels. On the other hand, H_2_O_2_ drastically increased intracellular ROS by ∼100 folds. Interestingly, co-treatment with GlyH-101 reduced H_2_O_2_-induced ROS formation by ∼80%. The same level of H_2_O_2_-induced ROS and degree of antioxidative protection by GlyH-101 were observed for at least 6 h after H_2_O_2_ challenges (data not shown). In addition, the level of intracellular GSH in erythrocytes was determined. As illustrated in Fig. 2B, GlyH-101 had no effect on GSH levels under basal (no H_2_O_2_ treatment) conditions. GSH contents in the absence and in the presence of GlyH-101 were 267.1 ± 6.8 nM/mg protein and 270.7 ± 6.7 nM/mg protein, respectively. Treatment of erythrocytes with H_2_O_2_ significantly (p<0.01) reduced GSH levels by ∼40% (level of GSH was 158.6 ± 7.3 nM/mg protein after treatment with H_2_O_2_). Of particular interest, the decrease in GSH induced by H_2_O_2_ was completely prevented by co-treatment with GlyH-101. The levels of GSH in H_2_O_2_ plus GlyH-101 group, which were not different from those under basal condition, were 257.6 ± 7.0 nM/mg protein.

### Effects of MK571 on ROS production and glutathione levels in erythrocytes

**Figure 3:**
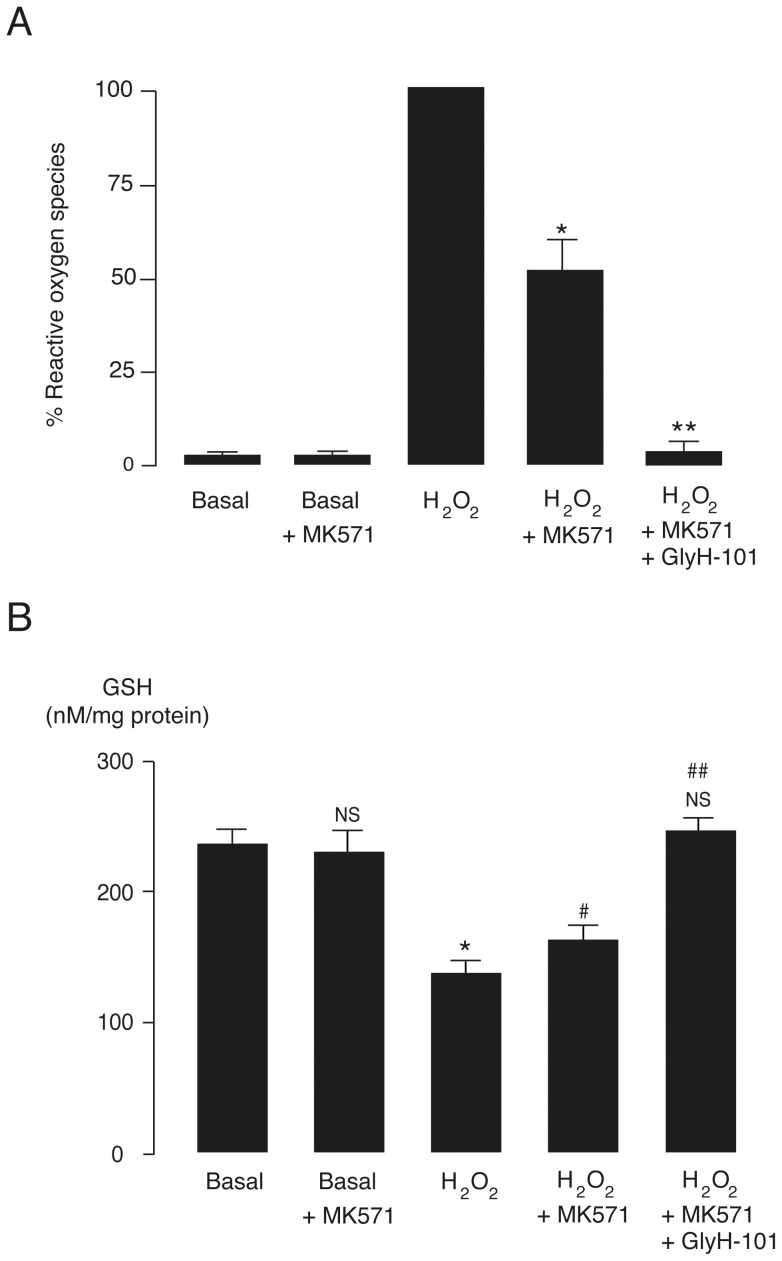
Anti-oxidative properties of MK571 in erythrocytes of beta thalassemia/Hb E patients. (A) MK571 reduced H_2_O_2_-induced free radical production. Erythrocytes loaded with DCFH-DA were incubated for 1 h with DMSO (basal), DMSO plus MK571 (basal + MK571), H_2_O_2_ (10 mM), H_2_O_2_ plus MK571 (50 µM) or H_2_O_2_ plus MK571 and GlyH-101 (50 µM). Reactive oxygen species were measured by flow cytometry analysis at excitation wavelength of 490 nm and emission wavelength of 530 nm. Data were expressed as means ± S.E. *, p<0.05; **, p<0.001 compared with H_2_O_2_-treated group (n = 12). (B) Effect of MK571 on intracellular glutathione levels. Erythrocytes were treated for 1 h without (basal) or with H_2_O_2_ (10 mM) in the presence or absence of MK571 (50 µM) or MK571 plus GlyH-101 (50 µM) before measurements of GSH levels. Data were expressed as means ± S.E. NS, non-statistical significant difference from basal control; *, p<0.01 compared with basal control; #, p<0.05; ##, p<0.01 compared with H_2_O_2_-treated group (n = 8).

Besides CFTR, MRP 1 has also been known to mediate glutathione effluxes in human erythrocytes [15]. Therefore, next sets of experiments were performed to investigate the effect of MRP 1 inhibitor, MK571, on ROS production and glutathione levels. It was found that MK571 (50 µM) reduced H_2_O_2_-induced ROS production by ∼50% (Fig. 3A). Interestingly, H_2_O_2_-induced ROS production was almost completely abolished by combined treatment with GlyH-101 and MK571. The results suggest that the effects of both inhibitors are additive. To determine whether the blockage of ROS production by MK571 is associated with the restoration of intracellular glutathione levels, the intracellular GSH level was assayed. Results showed that GSH levels were not altered by MK571, but were significantly decreased (p<0.01) after H_2_O_2_ challenges (Fig. 3B). GSH levels were 237.0 ± 11.1 nM/mg protein under basal conditions and 138.2 ± 7.9 nM/mg protein under H_2_O_2_-treated conditions. Treatment with MK571 significantly attenuated (p<0.05) the H_2_O_2_-induced decrease in GSH levels, which were 163.3 ± 11.0 nM/mg protein. Interestingly, combined treatment with GlyH-101 and MK571 completely abolished the effect of H_2_O_2_ on erythrocyte GSH levels (248.45 ± 7.28 nM/mg protein).

### Effect of GlyH-101 and MK571 on oxidative stress-induced glutathione efflux from erythrocytes

**Figure 4:**
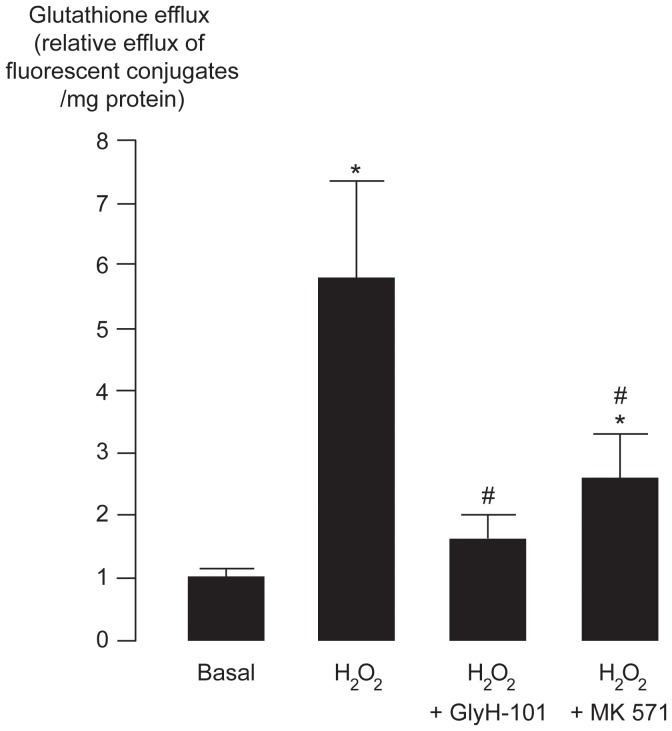
Effect of GlyH-101 and MK571 on H_2_O_2_-induced glutathione efflux. Erythrocytes loaded with CMFDA were incubated for 1 h with DMSO (basal), H_2_O_2_ (10 mM), H_2_O_2_ plus GlyH-101 (50 µM) or H_2_O_2_ plus MK571 (50 µM). Efflux of the fluorescent MF-SG extruded into the extracellular media over 1-h period of treatments was computed to indicate glutathione efflux. Data were expressed as mean relative efflux of fluorescent conjugates ± S.E. *, p<0.05 compared with basal control; #, p<0.05 compared with H_2_O_2_-treated group (n = 3).

To determine the degree of glutathione efflux inhibition by GlyH-101 and MK571, glutathione efflux from the erythrocytes was estimated using CMFDA-based efflux assays. Inside the cells, CMFDA releases its acetate groups and becomes a cell-impermeable fluorescent product CMF, which can readily react with intracellular glutathione forming a fluorescent glutathione conjugate MF-SG. In this assay, the amount of MF-SG extrusion (with hemolysis correction) into the extracellular media over 1-h period of treatments was measured as an index of glutathione efflux. As depicted in Fig. 4, H_2_O_2_ (10 mM) exposure caused a 5.7-fold increase in glutathione efflux from the erythrocytes. Co-treatment with GlyH-101 (50 µM) and MK571 (50 µM) significantly suppressed (p<0.05) the H_2_O_2_-induced glutathione efflux by 87% and 66%, respectively.

### Effect of GlyH-101 on oxidative stress-induced osmotic resistance of erythrocytes

**Figure 5:**
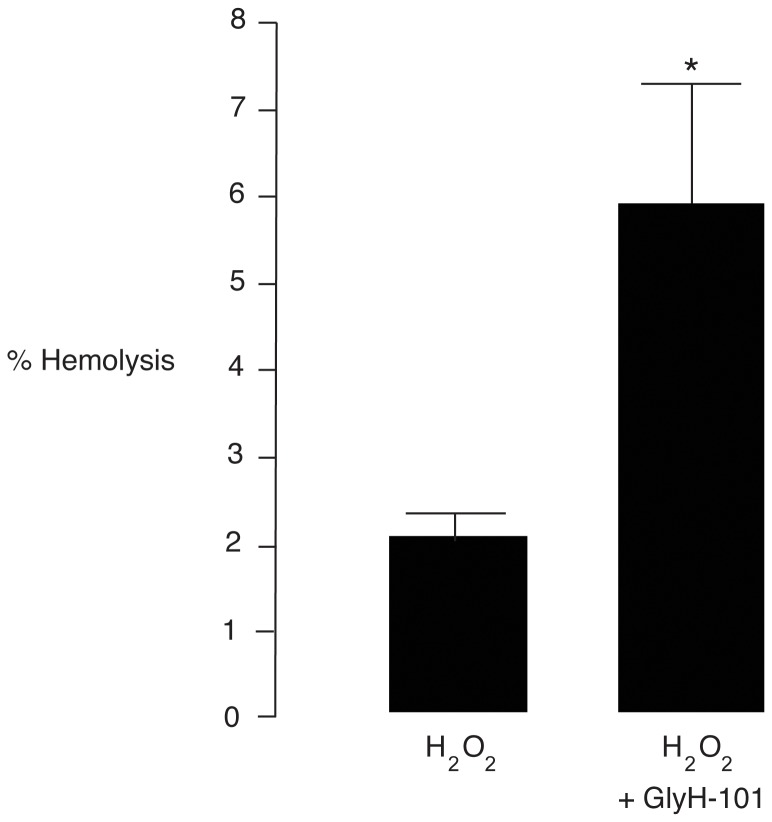
Effect of GlyH-101 on H_2_O_2_-induced osmotic tolerance of beta thalassemia/Hb E erythrocytes. The erythrocytes were incubated with PBS containing H_2_O_2_ (10 mM) with or without GlyH-101 (50 µM) at 37°C for 3 h in a shaking incubator. After centrifugation and removal of supernatant, cells were exposed to hypotonic solution (85% PBS) and centrifuged at 1,500 g at 4°C for 10 min. Absorbance of the supernatants was measured at 540 nm. Data were expressed as means ± S.E. *, p<0.05 compared with H_2_O_2_-treated group (n = 10).

It is known that oxidative stress induces phosphatidylserine externalization and shedding, resulting in increased cholesterol/phospholipid ratio and increased osmotic resistance of erythrocytes, which leads to the ultimate pathological events of suicidal cell death (or eryptosis) and premature destruction of the erythrocytes [27]. According to Freikman et al. [27], a reduction in hemolysis of erythrocytes after hypotonic solution challenge is considered as an indicator of oxidative stress-induced erythrocyte aging. We, therefore, measured hemolysis of beta thalassemia/Hb E erythrocytes induced by 85% PBS after exposure to H_2_O_2_ (10 mM) with or without GlyH-101 (50 µM). As shown in Fig. 5, percent hemolysis of H_2_O_2_-treated erythrocytes was only 2.0 ± 0.3% after exposure to hypotonic solution. However, this was increased to 5.9 ± 1.3% (p<0.05) by co-treatment with GlyH-101. The results indicate that GlyH-101 reduced the H_2_O_2_-induced senescence of thalassemia erythrocytes.

## Discussion

Oxidative stress in erythrocytes plays an important role in the pathogenesis of anemia in beta thalassemia/Hb E patients. Elevated oxidative stress accelerates senescence, enhances the rate of eryptosis and hence shortens the life span of erythrocytes. Antioxidant therapy has been shown to reduce serum oxidative stress and to increase the numbers of circulating erythrocytes in thalassemia patients [22], [28]. It is, therefore, expected to be of potential benefit in alleviating the severity of anemia in thalassemia patients. The present study showed that GlyH-101 and MK571, inhibitors of CFTR and MRP1, respectively, reduced oxidative stress-induced senescence of beta thalassemia/Hb E erythrocytes by inhibiting glutathione efflux.

We found that challenges of the beta thalassemia/Hb E erythrocytes with H_2_O_2_ caused a decrease in intracellular glutathione levels with a concomitant increase in glutathione efflux. This result indicates that oxidative stress induces glutathione efflux, a phenomenon previously described to precede the oxidative stress-induced apoptotic cell death in several types of cells such as astrocytes, endothelial cells and epithelial cells [6], [13], [14], [17]. In fact, it has been shown that circulating erythrocytes of thalassemia patients have lower GSH and total glutathione (GSH + GSSG) levels [29]. In addition, the oxidative challenge has been demonstrated to induce the MK571-inhibitable glutathione efflux from erythrocytes of healthy subjects [30], [31]. However, the oxidative stress-induced glutathione efflux and its transport mechanisms have never been investigated in erythrocytes of thalassemia patients. Inhibition by GlyH-101 and MK571 of H_2_O_2_-induced glutathione efflux indicates that both CFTR and MRP1 serve as major glutathione efflux pathways under the oxidative stress in beta thalassemia/Hb E erythrocytes.

It has been shown that oxidative stress induces increased calcium influx in erythrocytes, which, in turn, causes phosphatidylserine externalization and shedding, followed by eryptosis and destruction of erythrocytes by macrophages [27]. Oxidative stress is, therefore, considered as a central player in the pathogenesis of anemia in beta thalassemia/Hb E patients. Based on our findings that both GlyH-101 and MK571 reduced ROS production and ameliorated oxidative stress-induced membrane alterations in beta thalassemia/Hb E erythrocytes, GlyH-101, MK571 and other glutathione efflux inhibitors have potential therapeutic applications in beta thalassemia/Hb E, which may reduce the severity of anemia and other manifestations associated with increased erythrocyte destruction such as iron overload and splenomegaly. To provide evidence supporting this notion, further studies in the transgenic mouse model of thalassemia are required to demonstrate *in vivo* efficacy of the antioxidant effect of these compounds.

In summary, this study was the first to demonstrate that glutathione efflux inhibition reduces oxidative stress as well as its consequences in beta thalassemia/Hb E red cells. Further research and development on erythrocyte glutathione efflux inhibitors may provide novel therapeutic interventions for alleviation of oxidative stress-associated clinical abnormalities in beta thalassemia/Hb E patients such as anemia and iron overload.
